# The photo-transferred thermoluminescence phenomenon in case of emergency dose assessment

**DOI:** 10.1007/s00411-020-00834-1

**Published:** 2020-02-22

**Authors:** Małgorzata Wrzesień, Hiba Al-Hameed, Łukasz Albiniak, Joanna Maciocha-Stąporek, Michał Biegała

**Affiliations:** 1 Faculty of Physics and Applied Informatics, Department of Nuclear Physics and Radiation Safety, University of Lodz, Pomorska 149/153, 90-236 Lodz, Poland; 2 College of Science for Women, Department of Physics, University of Baghdad, Baghdad, Iraq; 3 Faculty of Biomedical Sciences and Postgraduate Training, Department of Medical Imaging Technology, Medical University of Lodz, Lindleya 6, 90-131 Lodz, Poland; 4 Department of Medical Physics, Copernicus Memorial Hospital in Lodz Comprehensive Cancer Center and Traumatology, Pabianicka 62, 93-513 Lodz, Poland

**Keywords:** PTTL, Emergency dose assessment, UV, Thermoluminescence

## Abstract

A major disadvantage of dose reconstruction by means of thermoluminescence (TL) is the fact that during readout of any TL material exposed to ionizing radiation (i.e., during measuring the glow curve), the radiation-induced signal gets lost. Application of the photo-transferred thermoluminescence phenomenon (PTTL) may offer a solution to this problem. In PTTL, the residual signal that is not destroyed by conventional TL readout (because it comes from deeper electron traps) can be readout through simultaneous stimulation by UV light and heating, allowing to obtain information about the absorbed dose in a second run. The present paper describes the application of PTTL for emergency dose assessment. For this, MTS-N thermoluminescent detectors (LiF: Mg, Ti) were exposed using a high-energy Clinac 2300 medical linear accelerator to doses of 100 mGy, 300 mGy, 500 mGy, 700 mGy and 1000 mGy. Irradiation with UV radiation allowed the determination of the optimal heating time of 3 h, while the optimal temperature was identified to be 70 °C. The results obtained demonstrated the usefulness of the PTTL method for emergency dose assessment. The efficiency of the PTTL method was determined as 19%. Finally it was found that the detector background after UV exposure should not be underestimated during routine dose measurements.

## Introduction

Thermoluminescent detectors (TLDs) made of lithium fluoride (LiF: Mg, Ti—MTS-N) and produced by Radcard in Kraków, Poland, are used in individual dosimetry of workers occupationally exposed to ionizing radiation. The range of doses of ionizing radiation that can be measured using such detectors ranges from 0.1 mSv to 10 Sv (Woźniak et al. 2006). The biggest problem associated with their use is that the readout procedure required to obtain information on the dose that the detector has been exposed to empty the electron (donor) traps, eliminates the collected information.

The phenomenon of photo-transferred thermoluminescence (PTTL) (Alexander et al. 1997; Alexander and McKeever 1998; Wintle and Murray 1997; Muñiz et al. 1999; Sas-Bieniarz et al. 2014) opened new possibilities for TLDs. In 1998, Alexander and McKeever described the photo-transferred thermoluminescence (PTTL) effect, where, after a standard reading, the TLD is exposed to UV radiation and read again. This effect can be explained by the presence of deeper TL traps that cannot be emptied by a standard reading. The following UV exposure makes the electrons in the TLD material migrate from deeper traps to shallower ones, allowing the TLD to be read again and the dose to be estimated a second time. The PTTL method for TLD-100 detectors was developed and used for personal dosimetry by Muñiz et al. (1999) and Delgado et al. (1996). The Institute of Nuclear Physics of the Polish Academy of Sciences (IFJ PAN) in Kraków, Poland, has developed a simple and convenient method which enables to re-estimate the dose in the range from 5 to 50 mGy using MTS-N TLDs, by means of the PTTL phenomenon (Budzanowski et al. 2013; Sas-Biernarz et al. 2014).

The present publication describes the application of the PTTL phenomenon to reassess doses ranging from 100 to 1000 mGy. This dose range was chosen because it is relevant in emergency situations. Readout of high doses requires correction of the settings of a manual TL reader, because the standard readout of a detector that registered a dose well above 100 mGy can result in an underestimation of the number of counts registered by the detector (Biegała 2003; International Standard 1991). This is so because a photomultiplier might saturate if the readout of dose is too high while the TEST parameter (as defined in a manual user for reader RA ’04 (RA ’04 Reader, Analyser TLD 2004)) is too low. In this case, the use of the PTTL phenomenon can be used to obtain at least partial information about the original dose the detector was exposed.

## Materials and methods

In the present study, 100 MTS-N TLDs (LiF: Mg, Ti) (Waligórski et al. 1999) manufactured by Radcard in Kraków, Poland, were used. Before the exposure, the detectors were annealed according to the manufacturer’s instructions, i.e. for 1 h at 400 °C in a Magma Therm MT 1105-E4 device produced by Magma Therm (Istanbul, Turkey), and for 2 h at 100 °C in a SUP-18 W dryer by Wamed (Warszawa, Poland).

The detectors were irradiated using the high-energy medical linear accelerator Clinac 2300, manufactured by Varian Medical Systems (Palo Alto, USA). During the irradiation, photons with a nominal energy of 15 MeV were used. All TLDs were placed in a PTW RW-3 constant phantom, equivalent to water for energies above 1.25 MeV, at a depth of 3 cm where the dose reached its maximum value. Irradiation was done using a square field with a size of 20 × 20 cm. The irradiation times were chosen in a way that the doses were 100 mGy, 300 mGy, 500 mGy, 700 mGy and 1000 mGy, respectively. The same dose values were used to expose the thermoluminescent detectors which were applied to take further measurements. A manual RA ’04 reader from Mikrolab (Kraków, Poland) was used to readout the TL signals induced in the TLDs.

After exposure and readout, the TLDs were exposed to UV radiation using an UVLMS-38 lamp produced by Analytik Jena US LLC (Upland, USA) which emitted three wavelengths of 254 nm, 302 nm and 365 nm. In the present paper a wavelength of 254 nm was used, because in previous studies no signal had been observed after exposure to UV radiation at 302 nm and 365 nm (Budzanowski et al. 2013). In this work, as well as in the one by Delgado et al. (1996) and Budzanowski et al. (2013), it was observed that the PTTL glow curve had a simple one-peak shape, dominated by peak V while peak IV was not present (Delgado et al. 1992; Budzanowski et al. 2013). During exposure to UV radiation, batches of 50 or 100 TLDs located on a copper plate with a size of 100.3 × 80.3 mm, were placed on a HC17.5D heating plate (CAT, Staufen, Germany) with a size of 125 × 125 mm. A similar procedure was applied to quantify the related background signals (Table 1).

**Table 1:** Subsequent steps of the measurement procedure

Step number	Description of the procedure	Motivation
PTTL signal analysis		
1	Annealing at 400 °C for 1 h, after that at 100 °C for 2 h	To delete the “memory” of the TLDs
2	Exposure to photon radiation with doses, ranging from 100 to 1000 mGy (20 TLDs/dose)	To record dose
3	Post-irradiation annealing at 100 °C	To erase the TL signal up to 100 °C
4	Readout with a heating rate of 5 °C/s	To obtain the TL signal
5	Exposure to ultraviolet radiation	To migrate electrons from deeper to shallower traps
6	Readout with a heating rate of 5 °C/s	To obtain the PTTL signal
Background analysis		
1	Annealing process of the detectors	To delete the “memory” of the TLDs
2	Readout with a heating rate of 5 °C/s	To obtain the TL signal
3	Exposure to ultraviolet radiation	To migrate electrons from deeper to shallower traps
4	Readout with a heating rate of 5 °C/s	To obtain the PTTL signal

*TL* thermoluminescence, *TLD* thermoluminescent detector, *PTTL* photo-transferred thermoluminescence

Exposure of TLDs to high doses of up to 1 Gy requires some corrections of the settings proposed in the manual of the RA ’04 reader, especially the parameter associated with the registration of photons emitted during the detector readout by the photomultiplier requires some corrections. The procedure specified below can be utilized to reassess the dose registered by the detector if—after a routine readout of a TLD—it turns out that the dose was high. This procedure requires determination of optimal temperature and heating time, which can be quantified during a re-reading of the MTS-N detector using ultraviolet light. More specifically, after exposure to ionizing radiation and readout, a TLD was placed on a heating plate for 2 h and exposed to UV radiation in the temperature range from 33° to 140 °C.

## Results and discussion

**Fig. 1:**
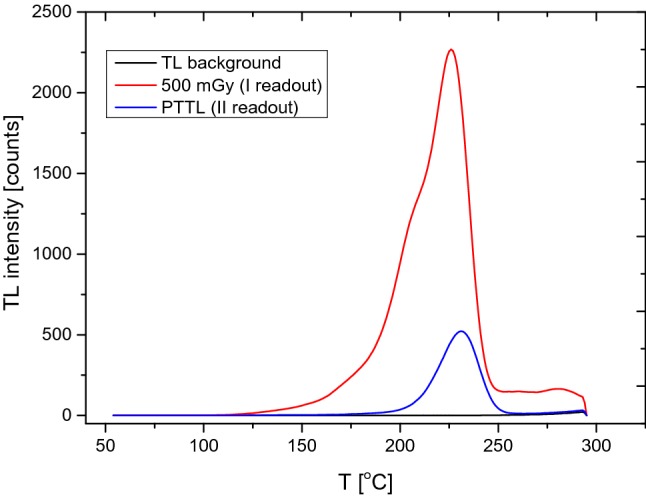
TL glow curves obtained for a MTS-N dosimeter irradiated with a dose of 500 mGy (red curve), then irradiated with UV radiation at 80 °C for 2 h (blue curve), and corresponding background glow curve (black curve**)**. *TL* thermoluminescence, *PTTL* photo-transferred thermoluminescence (color figure online)

Figure 1 shows, as an example, glow curves of a TLD obtained after exposure to ionizing radiation, followed by an exposure to UV radiation, and corresponding background glow curve. The MTS-N detector was exposed to ionizing radiation at a dose of 500 mGy. As in the work of Budzanowski et al. (2013), the maxima of both glow curves (after exposure to ionizing and UV radiation) appear at the same temperature, but the height of the PTTL glow curve is more than five times smaller compared to that of the glow curve after exposure to ionizing radiation.

**Fig. 2:**
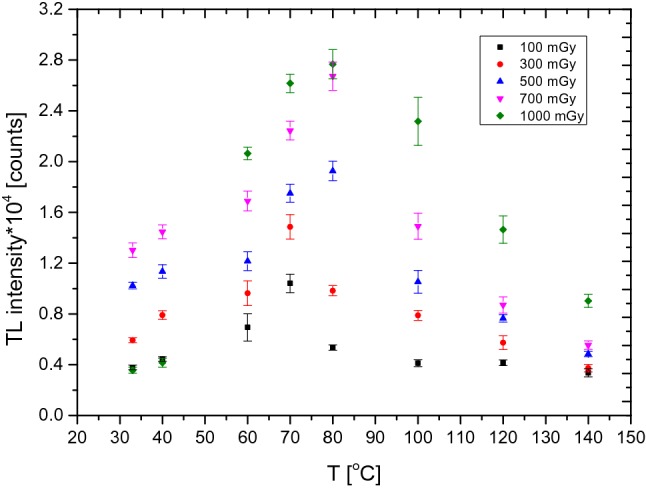
Relation between PTTL signal (an average value of ten PTTL signals with the standard error of the mean—SEM) and heating temperatures (ranging from 33° to 140 °C) during UV irradiation for 2 h (in each case). *TL* thermoluminescence

The procedure described above was used to determine the optimal temperature value that should be used before reading out the detector again. The results are shown in Fig. 2, which presents the average values obtained for ten PTTL signals including standard error of the mean. The optimal value of the temperature parameter depends on the ionizing radiation dose the TLD was exposed before. From Fig. 2 it follows that the temperature of heating of the detectors to obtain an optimal PTTL signal was 80 °C, for doses from 500 to 1000 mGy, while it was 70 °C for doses of 100 mGy and 300 mGy.

**Fig. 3:**
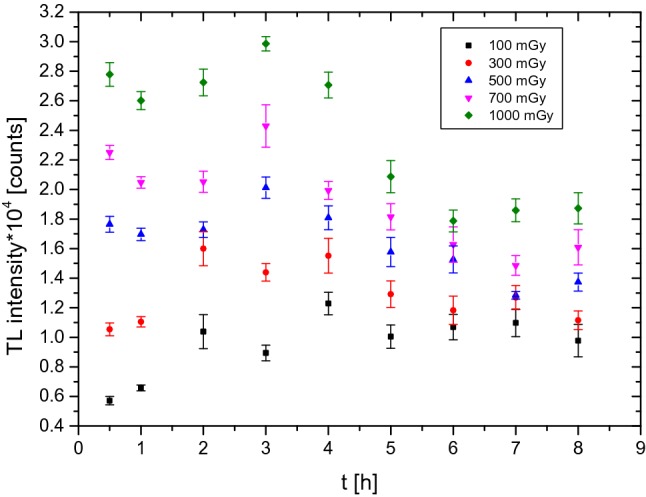
Relation between PTTL signal (an average value of ten PTTL signals with the standard error of the mean—SEM) and the heating time during UV irradiation at temperatures of 70 °C (for doses of 100 mGy and 300 mGy) and 80 °C (for doses in the range from 500 to 1000 mGy). *TL* thermoluminescence

To determine the optimal heating time for the detectors, the effect of the heating duration on the number of photons released during the reading out was investigated. The results, expressed as the average values of ten PTTL signals obtained after UV exposure between 0.5 h and 8 h, are presented in Fig. 3. For this experiment and in line with the results shown in Fig. 2, for doses of 100 mGy and 300 mGy, the heating temperature was set to 70 °C, while for doses in the range from 500 to 1000 mGy, detectors were heated to a temperature of 80 °C.

Figure 3 suggests that it is difficult to identify an optimal heating time for the whole range of investigated doses. Figure 3 shows that the maximum heating time, which is the time to obtain an optimal PTTL signal, is about 3 h for doses ranging from 500 to 1000 mGy, while it is about 2 h for a dose of 300 mGy, and about 4 h for a dose of 100 mGy.

**Fig. 4:**
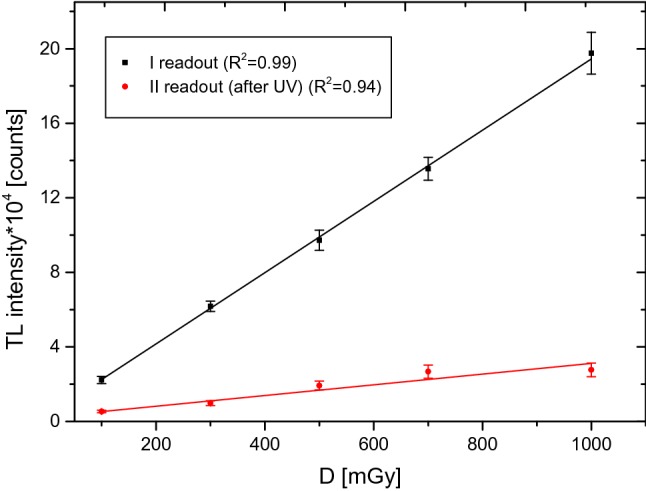
Relationship between the number of counts obtained from the glow curves after the first readout when the TLDs were exposed to doses in the range from 100 to 1000 mGy (black line), and that obtained after the second readout when the TLDs were exposed to UV at 80 °C for 2 h (red line). Measurement uncertainties—standard error of the mean (SEM). *TL* thermoluminescence (color figure online)

The maximum of the PTTL curve was considered as the basic criterion that determined the choice of the optimal parameters for the detector heating before the second readout (after the UV exposure had taken place). Due to the fact that the attempt to determine the optimal heating time and temperature did not give an unequivocal result, another selection criterion was introduced, i.e., the linearity of the detector responses as a function of dose. It is noted that the linearity with dose is the determining factor for a dosimeter to be used in routine individual dosimetry. Figure 4 presents the relationships between the number of counts obtained in the first readout (after the TLDs had been exposed to ionizing radiation) as a function of dose (dose range: 100–1000 mGy) and those obtained in the second readout (after 2 h of UV exposure at 80 °C). After the second readout, Budzanowski and co-workers reported a linear trend with dose in the 5–50 mGy dose range and predicted that such a linear trend should also be kept for higher doses Budzanowski et al. (2013). This is confirmed by Fig. 4.

**Fig. 5:**
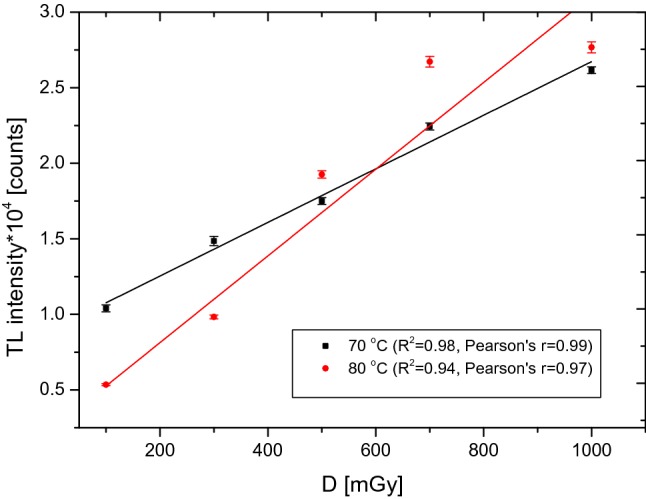
Relationship between the number of counts obtained from the glow curves after the second readout in the dose range from 100 to 1000 mGy, for an UV exposure of 2 h at 70 °C and 80 °C. Measurement uncertainties—standard error of the mean (SEM)

**Fig. 6:**
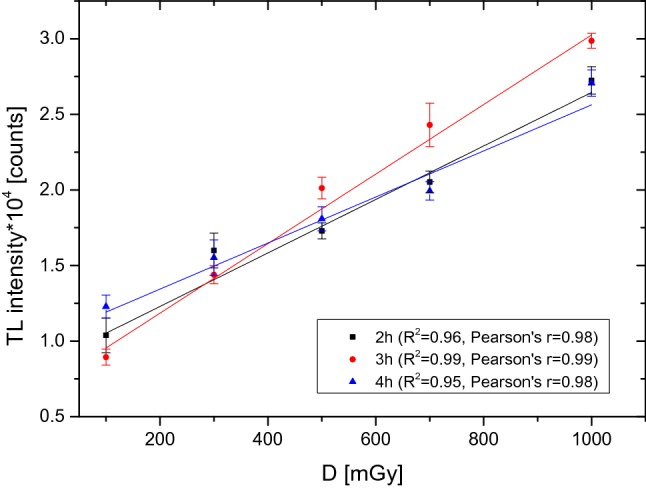
Relationship between the number of counts obtained from the glow curves after the second readout in the dose range from 100 to 1000 mGy, for an UV exposure of 2 h, 3 h and 4 h at 70 °C. Measurement uncertainties—standard error of the mean (SEM)

Figure 5 shows the dependence of the PTTL signal as a function of dose for two different temperatures, while Fig. 6 shows the PTTL signal for three different heating times, including the Pearson’s correlation coefficient for each curve. The value of this coefficient was used to identify the optimal temperature and heating time. As a result, the temperature of 70 °C appeared to be the most optimal one for the range of high doses of ionizing radiation, while the best linearity of the detector response after the second readout was obtained for a detector heating time of 3 h.

The efficiency of PTTL method for the applied range of ionizing radiation doses was also investigated. For this purpose, the ratio of the number of counts obtained after reading out a TLD irradiated with UV radiation and that obtained after its exposure to ionizing radiation was calculated. As a result, a ratio of about 19 ± 2% was achieved.

**Fig. 7:**
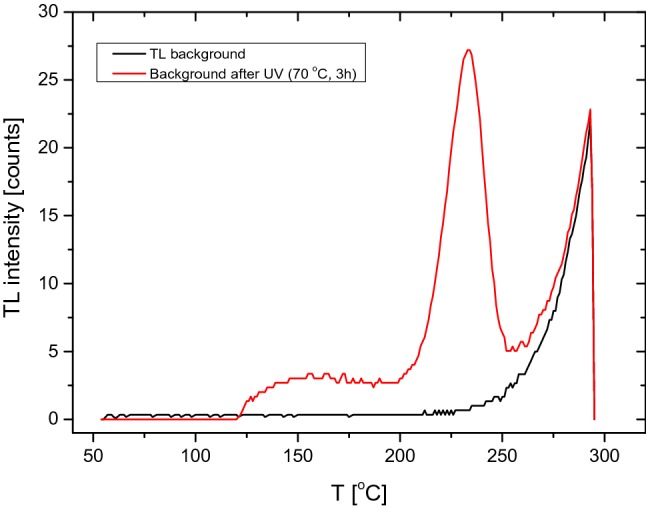
Background glow curves: first readout (black curve), second readout after UV exposure (red curve) (color figure online)

Abraham et al. in 2007 and other authors observed a high TL background signal after UV exposure (Abraham et al. 2007, 2008; Budzanowski et al. 2013; Bhasin et al. 1987). They also emphasized that it is necessary to subtract the high background before re-evaluating the dose. Therefore, both the TLD background and that after UV exposure were also investigated in the present paper. As an example, Fig. 7 shows the background glow curves obtained after first readout and after UV exposure. All these measurements were made using the optimal values for heating time and temperature. In half of the cases, the background after UV exposure was twice as high as the detector' background. This means that during routine measurements, the TLD background after UV exposure should not be underestimated.

## Conclusions

Emergency dose assessment, i.e. a situation in which the detector must be read again in the case of a thermoluminescent detector is particularly troublesome, because after a readout the TL information registered by the TLD is destroyed. In such cases, it might be useful to use the PTTL phenomenon to reassess the dose. In the present study, MTS-N TLDs were used and exposed to photon radiation using a high-energy Clinac 2300 medical linear accelerator with dose values in the range from 100 to 1000 mGy. Reassessment of the dose using the PTTL phenomenon requires determination of the UV wavelength that the detectors should be exposed to before re-reading out. The optimal wavelength was determined to be 254 nm. Temperature and heating time are, besides UV radiation, additional factors stimulating the phenomenon of PTTL. Consequently, these parameters were investigated in the present study, and it was found that the optimal temperature before re-reading was 70 °C, and the optimal heating time was determined to be about 3 h. Finally, the efficiency of the PTTL method was found to be 19 ± 2%. In half of the cases, there was a high TL background after exposure to UV radiation at 254 nm.

It is concluded that the use of the PTTL method in the case of emergency dose assessment is useful, because it means that the main disadvantage of thermoluminescent dosimetry becomes less important, which is why the method can represent a significant improvement in individual dosimetry (Muñiz et al. 1999).
